# Deep learning to convert unstructured CT pulmonary angiography reports into structured reports

**DOI:** 10.1186/s41747-019-0118-1

**Published:** 2019-09-23

**Authors:** Adam Spandorfer, Cody Branch, Puneet Sharma, Pooyan Sahbaee, U. Joseph Schoepf, James G. Ravenel, John W. Nance

**Affiliations:** 1 Department of Radiology, Medical University of South Carolina, 171 Ashley Avenue, Charleston, SC 29425 USA; 2 Siemens Medical Solutions USA, Inc., 40 Liberty Boulevard, Malvern, PA 19355 USA

**Keywords:** Artificial intelligence, Machine learning, Natural language processing, Structured reporting, Tomography (x-ray, computed)

## Abstract

**Background:**

Structured reports have been shown to improve communication between radiologists and providers. However, some radiologists are concerned about resultant decreased workflow efficiency. We tested a machine learning-based algorithm designed to convert unstructured computed tomography pulmonary angiography (CTPA) reports into structured reports.

**Methods:**

A self-supervised convolutional neural network-based algorithm was trained on a dataset of 475 manually structured CTPA reports. Labels for individual statements included “pulmonary arteries,” “lungs and airways,” “pleura,” “mediastinum and lymph nodes,” “cardiovascular,” “soft tissues and bones,” “upper abdomen,” and “lines/tubes.” The algorithm was applied to a test set of 400 unstructured CTPA reports, generating a predicted label for each statement, which was evaluated by two independent observers. Per-statement accuracy was calculated based on strict criteria (algorithm label counted as correct if the statement unequivocally contained content only related to that particular label) and a modified criteria, accounting for problematic statements, including typographical errors, statements that did not fit well into the classification scheme, statements containing content for multiple labels, etc.

**Results:**

Of the 4,157 statements, 3,806 (91.6%) and 3,986 (95.9%) were correctly labeled by the algorithm using strict and modified criteria, respectively, while 274 (6.6%) were problematic for the manual observers to label, the majority of which (*n* = 173) were due to more than one section being included in one statement.

**Conclusion:**

This algorithm showed high accuracy in converting free-text findings into structured reports, which could improve communication between radiologists and clinicians without loss of productivity and provide more structured data for research/data mining applications.

## Key points

- An artificial intelligence-based algorithm can be used to label statements from unstructured radiology reports, helping facilitate conversion into structured reports.
- Many statements were difficult to classify by both the manual observers and the algorithm, highlighting the limitations of free-form reporting.
- Based on the prediction probability for each statement, the algorithm could have utility in identifying ambiguous or otherwise problematic language in reports.

## Background

Clinical medical imaging involves a number of distinct components: proper image acquisition, reconstruction, interpretation, and reporting of findings. Traditionally, the body of the radiology report (*i.e.,* the “Findings” section) consists of unstructured, free-form dictations. However, this style has been found to result in ambiguous and difficult to interpret reports [1, 2], whereas more standardized reports result in improved accuracy and consistency of the radiologist [3, 4] along with improved satisfaction [2, 5], understanding [6], and recall [7] by the referring clinician. Unfortunately, widespread adaption of structured reports has met some resistance due to perceived compromises in workflow efficiency, potential oversimplification of findings, and concerns over professional billing [8–10].

While changes in traditionalist attitudes can be slow, advances in various artificial intelligence (AI) applications have been recently accelerating. Techniques combining natural language processing (NLP) and machine learning (ML) have been shown to have high accuracy in extracting content from unstructured radiology reports [11, 12], but there is a paucity of data applying these techniques to report restructuring. Accordingly, we sought to develop and validate a ML algorithm that is capable of consuming free-form computed tomography pulmonary angiography (CTPA) reports and generating structured reports. As a secondary goal, we sought to explore the utility of this algorithm in identifying problems with statements from the original reports.

## Methods

**Fig. 1:**
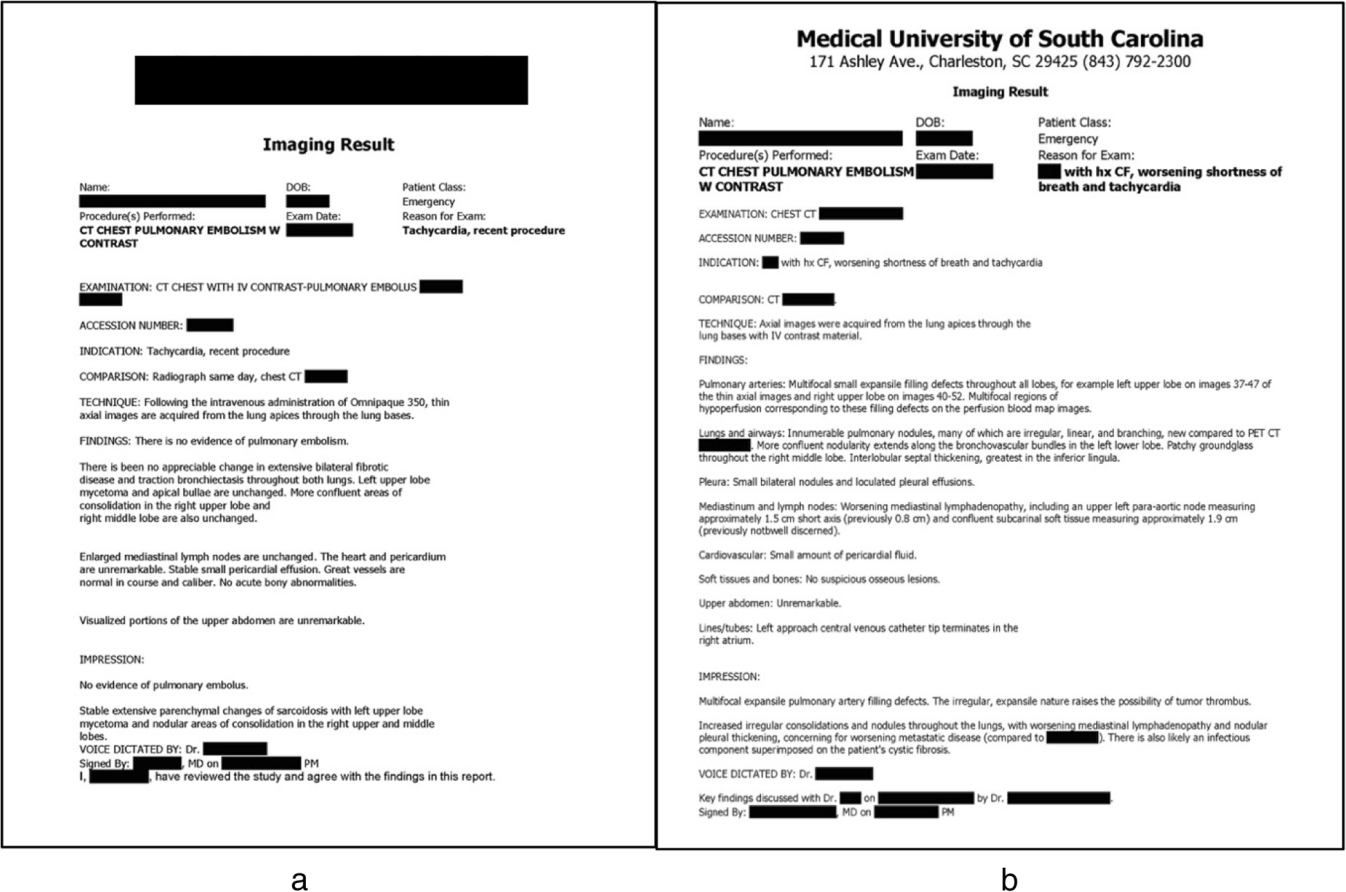
Examples of unstructured (**a**, used for testing) and structured (**b**, used for training) radiology reports

The Institutional Review Board approved and waived consent for this retrospective study. Our department moved from completely unstructured reporting of findings to the utilization of simple section headings in August 2016. Content within the unstructured reports was included under a general “Findings” section without any additional labeling; radiologists reported information in a traditional prose format (sentences organized into paragraphs, with no section headings). The structured reports included section headings within the findings section. Examples of each style are presented in Fig. 1.

### Algorithm design

**Fig. 2:**
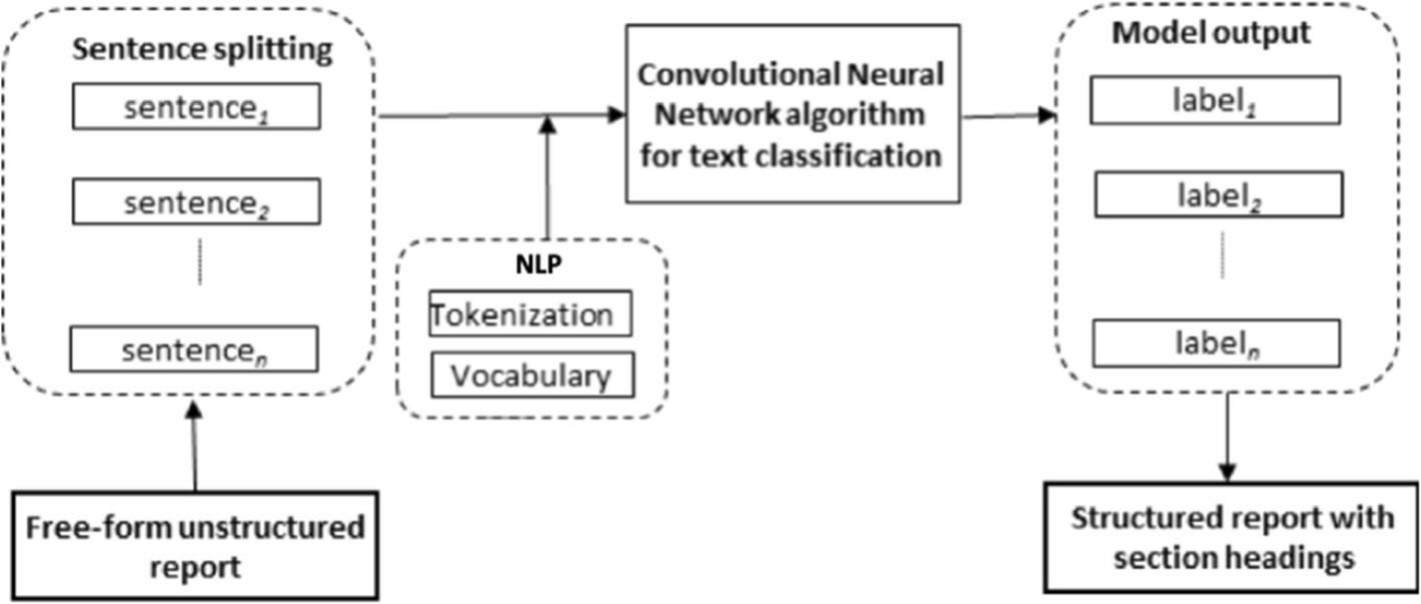
Overview of the proposed deep learning framework for converting free-form unstructured reports into structured reports with section headings. Each input report is split into sentences and each sentence is classified by the pre-trained convolution neural network algorithm into one of the classes (section headings). NLP, Natural language processing

A deep learning-based NLP framework was designed to automatically convert free-form reports into structured reports, as shown in Fig. 2.

The training data consisted of 475 structured CTPA reports (with section headings for all the findings) generated from November 2016 through April 2017. In the training data, each sentence was already annotated by a clinical expert since it was associated with a section heading in the structured report. The following sections headings were present: *Pulmonary arteries*; *Lungs and airways*; *Pleura*; *Mediastinum and lymph nodes*; *Cardiovascular*; *Soft tissues and bones*; *Upper abdomen*, and *Lines/tubes*. These annotated sentences were used as training samples.

To prepare the data for training, a pre-processing step was applied to each report, whereby each report was automatically split into sentences and words.

**Fig. 3:**
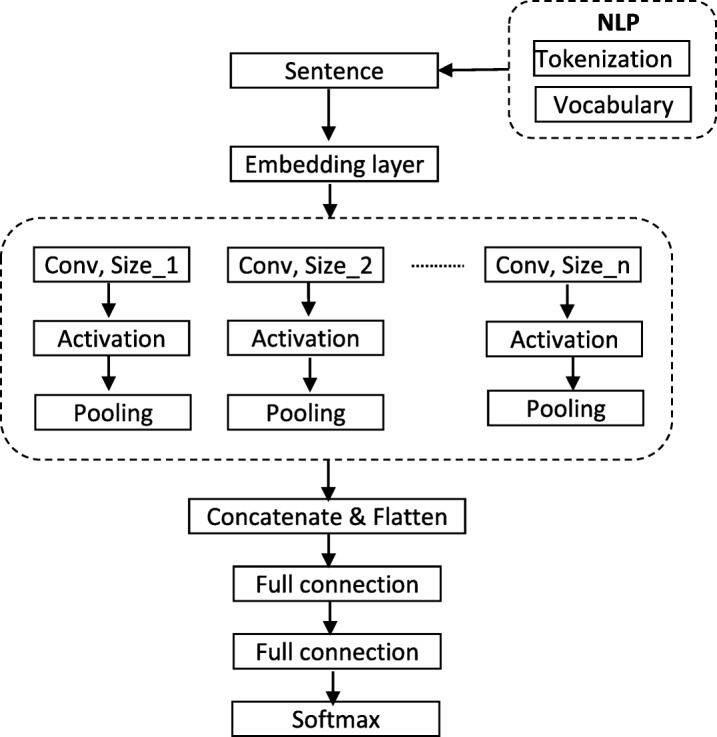
Text classification model with convolution neural network net. Conv, Convolution; NLP, Natural language processing

A convolutional neural network (CNN)-based text classification algorithm (Fig. 3), which operated on each sentence independently, was trained on the labeled data. The model input and output were a sentence and its corresponding section label, respectively. The word embedding layer in the network architecture converted each word into its vector representation. Due to limited data size, a pre-trained word embedding was used in this work. A “softmax” function in the last layer of the neural network (Fig. 3) provided probabilities for each label in the multi-class classification problem. The class with the highest probability was chosen as the predicted result.

### Algorithm validation

The validation data consisted of 400 unstructured CTPA reports (without section headings) generated from November 2015 through April 2016, with ground truth created by two human observers.

For the validation, each unstructured report was applied to the same pre-processing (sentence and word tokenization) as described above. Each sentence to be classified was tokenized with words, converted into word vectors, and fed to the pre-trained CNN model described above. The pre-trained model assigned a label to each input sentence. A structured report was generated when all sentences were labeled. A confidence score was generated by the algorithm for each prediction, ranging from 0 (least confident) to 1 (most confident).

All statements from the 400 unstructured test reports were classified by the ML algorithm into one of the eight sections as described above. For generating the ground truth, two independent human observers (one radiology resident and one radiology attending) manually placed each statement into one of the eight categories, if possible. Problematic statements, *i.e.,* those that did not fit well into one and only one of the eight predetermined labels, were identified and coded into one of seven general types of problems:

1. More than one section included in a single statement, for example, “There is no pleural or pericardial disease,” which applies to both the *Pleura* and *Cardiovascular* labels;
2. Findings are acceptable and variably placed in different sections, for example, hiatal hernias are reported in the *Upper abdomen* section by some in our practice and in the *Mediastinum and lymph nodes* section by others.
3. Statements related to technique or artifact, for example, “Study quality is severely degraded by patient motion artifact”;
4. Complicated cases that by necessity involve multiple sections, such as the following description of a lung cancer case: “5.1-cm mass in the right middle lobe with direct invasion of the mediastinum and encasement and narrowing of the right middle lobar pulmonary arteries,” which addresses components of *Pulmonary arteries*, *Lungs and airways*, and *Mediastinum and lymph nodes*;
5. Rare findings that are not well classified, for example, “Evidence of prior left hemidiaphragmatic repair”;
6. Typographical/dictation errors or nonsensical statements, for example, “Neuropathy material visualized within the midthoracic esophagus”;
7. Other.

The human observers had discretion on how to label problematic statements. Some received a single label (*e.g.,* image quality statements applying predominantly to the pulmonary arteries were given a label of *Pulmonary arteries*), some received multiple labels (*e.g.,* “There are no suspicious pulmonary nodules or mediastinal lymphadenopathy” would be given labels of *Lungs and airways* and *Mediastinum and lymph nodes*), and some received no label at all (*e.g.,* dictation errors/nonsensical statements: “Are no nodular”). Discrepancies between observers were resolved by consensus.

The observers then evaluated the accuracy of the predicted labels compared to the manual labeling, using two sets of criteria: “Strict” and “Modified.” The ML labels were considered correct by strict criteria if and only if they exactly matched the same label applied by the human observers. Accordingly, problematic statements could be considered correct by strict criteria if and only if the human observers placed a single label on the problematic statement and this matched the algorithmically derived label. In contrast, the ML labels were considered correct by modified criteria if they matched one of the labels applied by the human observers. For example, “There is no pleural or pericardial disease” would be coded as *Pleura* and *Cardiovascular* by the human observers. Either of these labels if predicted by the ML algorithm would be considered correct by modified criteria, but neither by strict criteria.

### Statistical analysis

All statistical analyses were performed using commercially available statistics software (SPSS v25, IBM Corp., Armonk, NY). Descriptive statistics utilized mean ± standard deviation or median with interquartile ranges.

## Results

Four-hundred reports were included in the test set, encompassing a total of 4,157 statements (10.4 ± 2.6 statements per report, mean ± standard deviation). Of the 4,157 statements, 3,806 (91.6%) were correctly labeled by the algorithm using strict criteria, while 3,986 (95.9%) were correctly labeled using modified criteria. The accuracy of individual labels using strict and modified criteria is shown in Table 1.

**Table 1:** Accuracy of individual predicted labels

Predicted label	Number of statements	Accuracy by strict criteria	Accuracy by modified criteria	Problematic statements
Cardiovascular	840	805/840 (95.8%)	815/840 (97.0%)	23/840 (2.7%)
Lines/tubes	118	111/118 (94.1%)	113/118 (95.8%)	2/118 (1.7%)
Lungs and airways	821	717/821 (87.3%)	768/821 (93.5%)	68/821 (8.3%)
Mediastinum and lymph nodes	447	402/447 (89.9%)	444/447 (99.3%)	48/447 (10.7%)
Pleura	371	307/371 (82.7%)	369/371 (99.5%)	62/371 (16.8%)
Pulmonary arteries	502	485/502 (96.6%)	487/502 (97.0%)	21/502 (4.2%)
Soft tissues and bones	583	553/583 (94.8%)	556/583 (95.4%)	16/583 (2.7%)
Upper abdomen	475	426/475 (89.7%)	434/475 (91.4%)	34/475 (7.2%)
Total	4,157	3,806/4,157 (91.6%)	3,986/4,157 (95.9%)	274/4,157 (6.6%)

### Problem statements

Of the 4,157 statements, 274 (6.6%) were problematic for the manual observers to label, of which 180 were misclassified using strict criteria. The causes for the problems were more than one section being included in one statement (*n* = 173, 4.2%), findings acceptable in more than one section (*n* = 38, 0.9%), statements related to technique or artifact (*n* = 28, 0.7%), typographical/dictation errors or nonsensical statements (*n* = 20, 0.5%), rare findings that are not well-classified into the predetermined schema (*n* = 11, 0.3%), and complicated cases that by necessity involve multiple sections for a single statement (*n* = 3, 0.1%). One statement referred to a prior examination and was considered problematic, subcategory *Other*. Problematic statements by label are shown in Table 1.

### Prediction probability

**Fig. 4:**
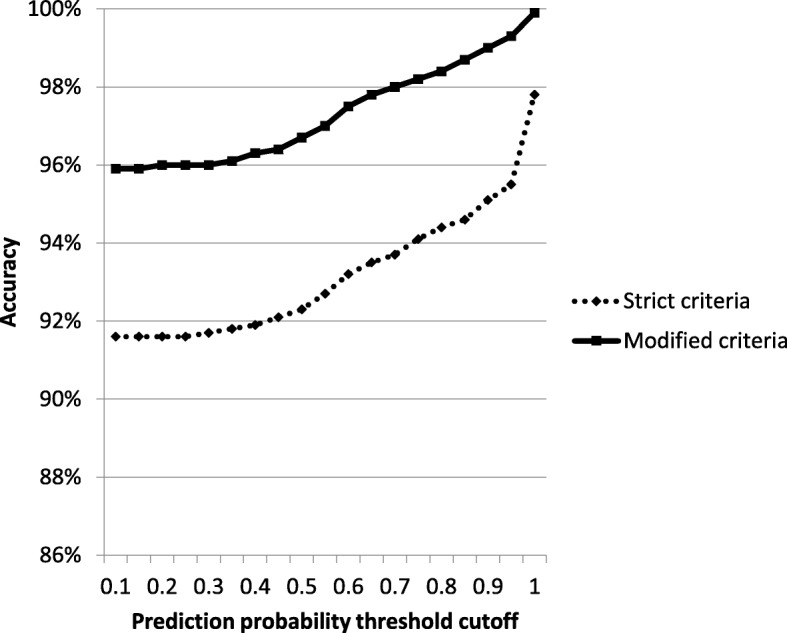
Accuracy of the algorithm in correctly labeling statements by strict and modified criteria at various prediction probability thresholds

The algorithm applied a prediction probability of 1.0 to the majority of statements (3,262/4,157, 78.5%); accordingly, the median and interquartile ranges were all 1.0 (1.0–1.0), whereas the total range of prediction probability for all statements was 0.146–1.000. The accuracy of the algorithm (using both strict and modified criteria) increased with increasing prediction probability (Table 2; Fig. 4). Of the 274 problem statements, 180 (65.7%) had a prediction probability < 1, compared to 71/3,883 (18.4%) statements without classification problems.

**Table 2:** Accuracy stratified by prediction probability

Prediction probability threshold	Number of statements	Accuracy by strict criteria	Accuracy by modified criteria
0.1	4,157	3806/4157 (91.6%)	3986/4157 (95.9%)
0.15	4,155	3806/4155 (91.6%)	3986/4155 (95.9%)
0.2	4,154	3806/4154 (91.6%)	3986/4154 (96.0%)
0.25	4,154	3806/4154 (91.6%)	3986/4154 (96.0%)
0.3	4,151	3805/4151 (91.7%)	3985/4151 (96.0%)
0.35	4,143	3802/4143, (91.8%)	3982/4143 (96.1%)
0.4	4,127	3794/4127 (91.9%)	3974/4127 (96.3%)
0.45	4,112	3786/4112 (92.1%)	3966/4112 (96.4%)
0.5	4,094	3779/4094 (92.3%)	3958/4094 (96.7%)
0.55	4,069	3771/4069 (92.7%)	3945/4069 (97.0%)
0.6	4,034	3761/4034 (93.2%)	3934/4034 (97.5%)
0.65	4,009	3747/4009 (93.5%)	3920/4009 (97.8%)
0.7	3,961	3711/3961 (93.7%)	3881/3961 (98.0%)
0.75	3,925	3693/3925 (94.1%)	3854/3925 (98.2%)
0.8	3,886	3667/3886 (94.4%)	3824/3886 (98.4%)
0.85	3,858	3651/3858 (94.6%)	3806/3858 (98.7%)
0.9	3,810	3623/3810 (95.1%)	3773/3810 (99.0%)
0.95	3,715	3546/3715 (95.5%)	3689/3715 (99.3%)
1.0	3,262	3187/3262 (97.8%)	3259/3262 (99.9%)

## Discussion

The current study demonstrated the feasibility of using an AI algorithm based on NLP and ML techniques to convert unstructured free-form text from the findings section of radiology reports into separate subheadings with high accuracy (92–96%). The results of the study also highlighted a well-known problem in radiology reporting: problematic statements, some of which pose difficulties to a structuring scheme by necessity (*e.g.,* complicated cases) and some by error (*e.g.,* dictation errors). While not designed for this purpose, the prediction probability feature of the algorithm may have an application in identifying such statements.

Clear, concise, accurate, and reproducible communication is a universally accepted requirement in the clinical practice of medical imaging [13], and both expert opinion and formal studies have shown that “structured reporting” in its various forms can improve communication between radiologists and referring providers [2, 3, 7, 13], albeit at the perceived cost of lower productivity [10, 13]. The current study demonstrated that new AI applications might be feasible in combining advantages of both free-form reporting (namely, increased radiologist productivity) and structured reporting (namely, improved communication with providers). In addition to immediate clinical benefits, increased structuring could have utility for data mining applications or when layering additional feature extraction on the report, and the algorithm could be retrospectively applied to legacy reports if indicated. For example, identification of the presence of pulmonary embolism via NLP and ML should be made easier if an algorithm only has to search a single section of a report (*Pulmonary arteries*) rather than the entire text.

Several different computer algorithms have been applied to radiology reporting in the past [11, 12, 14–16]. Studies have used a variety of different methods, *i.e.,* combinations of ML, active learning, and NLP. These studies have had varying goals, most commonly identification and highlighting specific findings, such as critical or abnormal findings [11, 14, 16] and presence of cancer [12], with wide ranges in diagnostic accuracies (range 82–99%) for the given task. Studies that are directly comparable to ours, *i.e.,* conversion of free-text reports to semi-structured reports, are sparse, but early feasibility studies have been promising [15].

Nearly 7% of the statements from the 400 test reports proved problematic for the manual observers to label. This highlights intrinsic problems in radiology reporting in general as well as some of the arguments against rigid report structuring. The majority of the problematic statements would not be inappropriate in a free-text report per se, *i.e.,* two sections combined into one statement or findings that could reasonably go into one of several headings, but rather became inconclusive when forced into one particular section. This could be reasonably addressed with either a change in dictation culture or more sophisticated rules layered onto the ML algorithm to address these specific situations. However, there were also a minority (0.5%) of statements that were nonsensical such that their meaning could not be determined enough for manual labeling, presumably from dictation errors or typos. Of these 20, only 3 (15%) received a prediction probability of 1 (meaning that the algorithm believed labeling was correct). Of all of the problematic statements, 66% had a prediction probability of less than 1, compared to only 18% of the nonproblematic statements. We believe that with modification, this feature of the algorithm could be used to identify statements within a report that should be reexamined for clarity. Of course, the algorithm was not designed with this feature in mind and the study was not designed to test such a feature, but we believe it warrants further examination.

This study is not without limitations, some of which highlight intrinsic challenges of ML technology. While over 4,000 individual statements were used for both training and testing, many times more will be necessary to optimize the potential of current ML algorithms, particularly in the accurate labeling of rare findings or uncommon statements. Both the training and testing reports were generated by approximately 40 separate radiologists, but all from the same department, and therefore both heterogeneity in individual reporting style and specific institutional nuances are incorporated into the algorithm. While it is likely a positive feature to have variability when training the algorithm, this did decrease labeling accuracy when applying our “strict” criteria. Conversely, institutional colloquialism could limit the generalizability of algorithms trained in a single radiology department. Our statement segmentation was based on individual sentences, which sometimes lacked the nuance available and necessary to convey information with prose-style dictations. It would potentially be more useful to look at word groupings rather than individual sentences when segmenting reports. Along the same lines, simply rearranging the order of statements from a prose-based report into different subsections might decrease the readability of the overall report. We would expect dictation styles to change with the implementation of this sort of algorithm (less contiguous prose and more individual statements). We only trained and tested our algorithm in English. Applying a “language translation algorithm” to non-English reports to convert to English or using multilingual word embedding might allow other languages to be tested and used. Finally, we must acknowledge that the definition of “structured reporting” varies. We acknowledge that this algorithm performed only the most basic of structuring—applying section labels. Other schemata also incorporate lexicons (*e.g.,* Breast Imaging Reporting and Data System, BI-RADS) in addition to section headings [17]; applying these more advanced techniques should be examined in future applications.

In conclusion, we demonstrated that an AI algorithm has high accuracy in converting free-text radiology findings into structured reports. This could improve communication between radiologists and referring clinicians without loss of productivity and provide more structured data for research/data mining applications. In addition, the prediction probability feature of the algorithm warrants further exploration as a potential marker of ambiguous statements.

## Abbreviations

AI: Artificial intelligence
CTPA: Computed tomography pulmonary angiography
ML: Machine learning
NLP: Natural language processing
